# Knowledge, attitudes, and practices of chemotherapy adverse reactions and care among patients with gastrointestinal lymphoma

**DOI:** 10.3389/fmed.2025.1722022

**Published:** 2026-01-23

**Authors:** Ping Li, Ping Zhang, Xuan Cui, Suqin Zhang, Hui Liu, Yujie Liu, Meitao Li

**Affiliations:** 1 Nursing Department, Peking University Cancer Hospital Inner Mongolia Hospital, Hohhot, Inner Mongolia Autonomous Region, China; 2 Oncology Department, Peking University Cancer Hospital Inner Mongolia Hospital, Hohhot, Inner Mongolia Autonomous Region, China; 3 Department of Gynecology and Oncology, Peking University Cancer Hospital Inner Mongolia Hospital, Hohhot, Inner Mongolia Autonomous Region, China; 4 Endoscopy Center, Peking University Cancer Hospital Inner Mongolia Hospital, Hohhot, Inner Mongolia Autonomous Region, China; 5 Thoracic Surgery, Peking University Cancer Hospital Inner Mongolia Hospital, Hohhot, Inner Mongolia Autonomous Region, China

**Keywords:** adverse reactions, chemotherapy, gastrointestinal lymphoma, health education, knowledge, attitude, and practice, socioeconomic status

## Abstract

**Purpose:**

To explore the knowledge, attitudes, and practices (KAP) regarding chemotherapy adverse reactions and care among patients with gastrointestinal lymphoma.

**Methods:**

This cross-sectional study was conducted between March, 2024, and May, 2024, at the Peking University Cancer Hospital Inner Mongolia Hospital, China. The participants included patients with gastrointestinal lymphoma. The KAP scores were collected using a researcher-developed questionnaire. The KAP levels were evaluated based on Bloom's cutoff value, and the associations among KAP were evaluated by logistic regression and structural equation modeling (SEM) analysis.

**Results:**

A total of 422 patients with gastrointestinal lymphoma participated in this study. The mean scores for knowledge, attitude, and practice were 12.93 ± 4.21 (range: 0–22), 27.57 ± 3.72 (range: 7–35), and 33.57 ± 3.19 (range: 8–40), respectively. The regression analysis indicated that knowledge scores significantly influenced attitudes (OR = 1.397, *P* < 0.001) and practice (OR = 1.235, *P* < 0.001). SEM analysis revealed that knowledge significantly influences practice behaviors directly (β = 0.161, *P* < 0.001) and indirectly through attitudes (β = 0.649, *P* < 0.001).

**Conclusion:**

Patients with gastrointestinal lymphoma demonstrated moderate knowledge, positive attitudes, and good practices regarding chemotherapy adverse reactions and care. Targeted interventions to improve knowledge, especially among rural and lower-income patients, may enhance overall attitudes and practices toward chemotherapy management.

## Background

Gastrointestinal (GI) lymphomas represent a significant subset of extranodal lymphomas, accounting for approximately 30%−40% of all extranodal non-Hodgkin lymphomas (NHL) (1). Although primary GI lymphoma (PGIL) is relatively rare, comprising only 1%−4% of all GI malignancies, its incidence has been steadily increasing over the past decades (2). A population-based study conducted in Israel by Dizengof et al. revealed significant data on the incidence rates of PGI-NHL (31). The study reported an overall age-adjusted incidence rate of 0.75 cases per 100,000 person-years (95% CI: 0.67–0.84) for primary GI non-Hodgkin's lymphoma (3). The distribution of PGIL varies across different anatomical sites within the GI tract. The stomach remains the most common site, accounting for 50%−60% of all cases, followed by the small intestine (30%−35%), and the colorectum (10%−15%) (4).

Recent advances in medical technology have significantly transformed the treatment landscape for GI lymphomas. Chemotherapy remains a cornerstone in the management of these malignancies, but its efficacy has been greatly enhanced by the integration of targeted therapies (5). However, despite its efficacy, chemotherapy is associated with a range of adverse reactions that can significantly impact patients' quality of life. Common side effects include nausea, vomiting, fatigue, myelosuppression, and neurotoxicity (6). These adverse reactions not only affect patient wellbeing but can also lead to dose reductions, treatment delays, or even premature discontinuation of therapy, potentially compromising treatment efficacy (7).

Given these challenges, there is a growing emphasis on effective management of chemotherapy-induced adverse reactions. Recent studies have highlighted the importance of proactive supportive care measures and patient education in mitigating these side effects (8). Because chemotherapy in gastrointestinal lymphoma carries a higher probability of serious complications related to the digestive tract, patients may require more specific knowledge and self-management skills to ensure timely recognition and response to adverse reactions (32, 33). Therefore, examining KAP in this population is particularly important. Improved management strategies have been shown to enhance patient compliance, reduce treatment interruptions, and ultimately improve overall outcomes (9).

In light of these developments, the focus of clinical research has increasingly shifted toward optimizing supportive care and developing interventions to better manage and alleviate chemotherapy-related toxicities. This approach aims to improve both the quality of life for patients undergoing treatment for GI lymphomas and the overall efficacy of chemotherapy regimens (10).

Knowledge, attitudes, and practices (KAP) surveys are employed in health behavior research to depict a specific study population and to collect information about what is known, believed, and practiced about scientific topics (11). The primary objective is to explore prevalent issues in current practice and offer valuable insights to direct the improvement of health education and disease management among diverse populations (12). The management of adverse reactions to chemotherapy can influence the control of such reactions and the overall recovery of patients through the combined efforts of patients, family members, and staff (7). This encompasses the extent of patients' grasp of chemotherapy adverse reaction and nursing knowledge, the degree of their positive attitude, and the implementation of practical nursing measures. Recent studies have highlighted the increasing importance of patient education and engagement in managing chemotherapy side effects (13). Insufficient knowledge of adverse reactions to chemotherapy can critically undermine treatment adherence and delay recognition of side effects, potentially exacerbating patient outcomes. This lack of awareness may lead to patient noncompliance, either through discontinuation of therapy or non-adherence to prescribed regimens, increasing the likelihood of suboptimal treatment responses and reduced survival rates (14). Thus, educational interventions are essential to enhance patient understanding and engagement, thereby improving clinical outcomes in chemotherapy settings. However, there is no systematic study on knowledge-attitude-practice of patients. The objective of this study was to examine the current KAP of patients diagnosed with GI lymphoma concerning adverse reactions to chemotherapy and nursing care, and to identify the influencing factors.

## Methods

### Study design and participants

This cross-sectional study was carried out prospectively between March, 2024, and May, 2024, at the Peking University Cancer Hospital Inner Mongolia Hospital, China. The hospital is a tertiary grade A specialized cancer hospital that integrates medical care, teaching, research, rehabilitation, cancer prevention, and health services. The hospital has a sanctioned capacity of 900 beds and a staff complement of approximately 1,200 personnel. The participants consisted of patients with GI lymphoma, the lymphoma subtype was not restricted; both Hodgkin lymphoma and NHL were included (15). The study protocol was ethically approved by the Ethics Committee of Inner Mongolia Hospital of Peking University Cancer Hospital (No. KY202465), and informed consent was obtained from all participants. The inclusion criteria were as follows: patients aged 18–75 years, a range chosen to minimize the impact of age-related frailty or cognitive decline on the reliability of questionnaire responses; those with clear consciousness and the ability to communicate effectively to ensure accurate questionnaire completion; and individuals receiving or planning to receive chemotherapy, as baseline KAP prior to treatment initiation is essential for understanding educational needs. The exclusion criteria included patients with severe malnutrition, cognitive impairment, psychiatric disorders, or comorbidities such as diabetes that might confound chemotherapy-related symptoms, as well as patients with non-GI lymphoma receiving chemotherapy. Individuals with religious beliefs that could influence their attitudes toward illness or treatment were also excluded to avoid potential confounding. No additional exclusion criteria were applied.

### Questionnaire and data collection

The questionnaire design was informed by professional clinical knowledge and key content related to the management of chemotherapy adverse reactions (15, 16). In addition to the cited literature, the development of questionnaire items was also informed by clinical experience in managing gastrointestinal lymphoma and commonly recognized nursing care practices related to chemotherapy. The overall structure and dimensions of the questionnaire followed the standard KAP theoretical framework. Following its development, a pilot study was conducted using 25 questionnaires, resulting in a Cronbach's α coefficient of 0.960. The questionnaire was developed and distributed in Chinese. The English version used for reporting underwent forward and backward translation for accuracy. The translation was performed by two authors with clinical and research experience in oncology to ensure terminological accuracy and conceptual equivalence. Face and content validation were conducted following established methodological procedures described in previous studies (17). During the pilot study, participants reported no difficulty in understanding the terminology used, and patients were allowed to seek clarification from healthcare staff whenever needed. Content validation was performed by three clinical experts with experience in gastrointestinal oncology and chemotherapy nursing, and all items were evaluated for correctness, relevance, and clarity; no revisions were required based on their feedback. In addition, Cronbach's alpha values for the individual sections were 0.9352 for the knowledge section, 0.9436 for the attitude section, and 0.9003 for the practice section. The pilot participants were patients with gastrointestinal lymphoma who met the same inclusion criteria as the main study, and no modifications to the questionnaire items were required based on the pilot findings.

The questionnaire comprised four distinct sections: (1) demographic characteristics; (2) knowledge dimension: eleven questions assessed participants' familiarity with GI lymphoma characteristics, treatment effectiveness, common adverse reactions to chemotherapy, nursing care measures during chemotherapy, and also recommended practice. These items were designed as self-evaluated knowledge measures, a commonly used approach in KAP studies to assess perceived familiarity with disease-related information (18). Although the study focused on chemotherapy adverse reactions, “care” was included because several items assessed patients' understanding of recommended nursing care measures during chemotherapy, which forms an integral part of safe and effective management. The scoring and classification approach for all three dimensions was based on Bloom's cut-off criteria. Responses were categorized as “Very familiar” (2 points), “Somewhat familiar” (1 point), or “Not familiar” (0 points). The total knowledge score ranged from 0 to 22 points. Knowledge levels were classified using Bloom's cut-off criteria, which are widely applied in KAP studies, and were categorized as low ( ≤ 13), moderate (14–17), or high (18–22) (19); (3) attitude dimension: seven questions employed a five-point Likert scale (Strongly agree to Strongly disagree) to gauge perceptions and attitudes toward chemotherapy adherence, adverse reactions, and treatment impact. Scores ranged from 7 to 35 points, with higher scores indicating more positive attitudes. Attitude scores were classified according to Bloom's cut-off criteria commonly used in KAP research, as negative ( ≤ 21), neutral (22–28), or positive (29–35) (19); and (4) practice dimension: eight questions assessed respondents' practices related to understanding and managing chemotherapy side effects, dietary adjustments, and treatment persistence. Responses were rated on a five-point frequency scale from “Always” (5 points) to “Never” (1 point). The total practice score ranged from 8 to 40 points. Practice levels were categorized following Bloom's cut-off criteria widely applied in KAP studies, as poor ( ≤ 24), fair (25–32), or good (33–40) (19). An overall KAP score was calculated by summing the scores from all three dimensions, ranging from 15 to 97 points. Overall KAP levels were classified using Bloom's cutoff as low ( ≤ 58), moderate (59–77), or high (78–97) (19).

The questionnaire was distributed through multiple channels, including WeChat groups, QR code scanning, and on-site clinical distribution. To prevent duplicate or invalid submissions, all completed electronic questionnaires were reviewed during the statistical analysis process, and entries submitted within an extremely short completion time (less than 120 s) were excluded. In addition, the online system restricted each device to a single submission, and manual data screening was performed to identify and remove any potential duplicate entries. The questionnaire was self-administered electronically, and participants completed and submitted it through an online platform. Informed consent was obtained on the first page of the e-questionnaire prior to participation.

### Sample size calculation

The required sample size was estimated using Cochran's formula for cross-sectional studies: *n* = *Z*^2^ × *p* × (1 – *p*)/*e*^2^. In this study, *Z* was set at 1.96 for a 95% confidence level, *p* was set at 0.5 to ensure the maximum required sample size (as no previous study has reported KAP in this topic), and the margin of error *e* was set at 0.05. Based on this calculation, the minimum sample size required was 384. Considering potential non-response, a total of 422 participants were ultimately included.

### Statistical analysis

The statistical analysis was conducted using R version 4.3.2 and Stata 18.0. The continuous variables, scores for KAP dimension, were tested for normality using Shapiro-Wilk test. Data conforming to normal distribution were expressed using the mean ± standard deviation (SD), and compared by ANOVA or independent-samples *t*-test. Data conforming to skewed distribution were expressed by the median, 25th percentile, and 75th percentile. The categorical variable, including responses to individual questions and demographic characteristics, were presented as *n* (%).

Univariable and multivariable logistic regression were used to explore factors independently associated with KAP scores. The scores in KAP dimensions were converted to categorical variables according to their median. Characteristics with a *P* < 0.1 in univariable regression were incorporated into the multivariable regression. This threshold is commonly used in exploratory analyses to ensure that potentially relevant variables are not prematurely excluded from multivariable modeling (34). Leveraging the KAP theoretical framework, a structural equation model (SEM) was applied to examine whether attitudes mediated the relationship between knowledge and practice behaviors. Both indirect and direct effects were calculated and compared, with model fit indices evaluated based on RMSEA < 0.08, SRMR < 0.08, TLI > 0.8, and CFI > 0.8. A two-sided *P* < 0.05 was considered as statistical significance. Multivariable logistic regression models were used to identify factors associated with each KAP dimension. For SEM, model assumptions were evaluated through standard diagnostic checks, including assessment of multivariate normality, examination of residuals, and evaluation of overall model fit indices.

## Results

### Demographic characteristics

The study included 422 patients with GI lymphoma. The mean age was 52.7 years, 61.8% were male, and the majority (64.5%) lived in cities. Most patients (93.6%) were married, and the patients had different educational backgrounds (primary school or below: 31.8%; middle school: 32.2%; high school or technical secondary school: 16.6%, and associate degree or above: 19.4%). The distribution of monthly income was as follows: 42.2% less than 2,000 yuan, 43.1% between 2,000 and 5,000 yuan, and 14.7% more than 5,000 yuan. Most participants (97.2%) had social health insurance. Regarding the disease characteristics, 63.7% of the patients had been diagnosed for less than 1 year, 27.0% for between 1 and 3 years, and 9.2% for more than 3 years. Currently, 53.8% of the patients were receiving chemotherapy. The remaining patients had not yet initiated chemotherapy at the time of the survey but were scheduled to receive it, and their responses reflect baseline KAP prior to treatment initiation (Table 1).

**Table 1:** Demographic characteristics and KAP scores.

**Demographic characteristic**	***N* (%)**	**Knowledge**	***P***	**Attitude**	***P***	**Practice**	***P***
		**Mean** ±**SD**		**Mean** ±**SD**		**Mean** ±**SD**	
Overall population	422 (100.0)	12.93 ± 4.21		27.57 ± 3.72		33.57 ± 3.19	
**Gender**
Male	261 (61.8)	12.76 ± 4.27	0.207	27.28 ± 3.76	0.030	33.39 ± 3.24	0.219
Female	161 (38.2)	13.20 ± 4.10		28.04 ± 3.62		33.86 ± 3.08	
**Age, years old**
18–49	69 (16.4)	14.10 ± 5.33	0.081	28.57 ± 4.00	0.052	34.62 ± 3.34	0.070
50–64	146 (34.6)	12.47 ± 4.03		27.36 ± 3.70		33.23 ± 3.63	
65–74	157 (37.2)	12.87 ± 3.64		27.30 ± 3.57		33.46 ± 2.56	
≥75	50 (11.8)	12.86 ± 4.44		27.64 ± 3.75		33.46 ± 3.14	
**BMI, kg/m** ^2^
< 18.5	45 (10.7)	13.07 ± 4.63	0.918	28.04 ± 3.94	0.633	34.11 ± 2.99	0.527
18.5–23.9	218 (51.7)	12.92 ± 4.06		27.51 ± 3.61		33.44 ± 3.02	
24.0–27.9	117 (27.7)	12.78 ± 4.37		27.52 ± 3.91		33.60 ± 3.77	
≥28.0	42 (10.0)	13.29 ± 4.13		27.48 ± 3.59		33.60 ± 2.40	
**Residence**
Rural	150 (35.5)	12.51 ± 4.07	0.011	27.35 ± 3.73	0.255	33.22 ± 2.77	0.059
Urban	272 (64.5)	13.16 ± 4.27		27.69 ± 3.72		33.77 ± 3.38	
**Marital status**
Married	395 (93.6)	12.81 ± 4.14	0.028	27.46 ± 3.72	0.008	33.51 ± 3.16	0.235
Unmarried/divorced/widowed	27 (6.4)	14.74 ± 4.82		29.15 ± 3.49		34.44 ± 3.49	
**Education**
Primary school or below	134 (31.8)	12.75 ± 3.94	< 0.001	27.37 ± 3.92	0.004	33.61 ± 2.90	0.029
Middle school	136 (32.2)	12.06 ± 3.57		26.96 ± 3.26		32.99 ± 2.58	
High school/technical secondary school	70 (16.6)	13.89 ± 3.87		27.96 ± 3.61		33.99 ± 3.03	
Associate or above	82 (19.4)	13.85 ± 5.43		28.59 ± 4.02		34.13 ± 4.36	
**Monthly income, yuan**
< 2,000	178 (42.2)	12.07 ± 3.82	< 0.001	26.67 ± 3.53	< 0.001	32.91 ± 2.70	< 0.001
2,000–5,000	182 (43.1)	13.20 ± 3.93		27.97 ± 3.60		33.86 ± 2.80	
>5,000	62 (14.7)	14.63 ± 5.35		28.97 ± 4.03		34.65 ± 4.78	
**Medical insurance**
Have social health insurance	410 (97.2)	12.86 ± 4.11	0.081	27.48 ± 3.70	0.006	33.52 ± 3.15	0.154
No social health insurance	12 (2.8)	15.42 ± 6.49		30.50 ± 3.40		35.33 ± 3.96	
**Diabetes**
No	354 (83.9)	12.83 ± 4.18	0.263	27.48 ± 3.76	0.200	33.47 ± 3.22	0.136
Yes	68 (16.1)	13.44 ± 4.32		28.03 ± 3.50		34.13 ± 2.96	
**Hyperlipidemia**
No	297 (70.4)	13.06 ± 4.26	0.311	27.64 ± 3.79	0.551	33.63 ± 3.38	0.590
Yes	125 (29.6)	12.62 ± 4.07		27.41 ± 3.57		33.45 ± 2.68	
**Time of diagnosis of gastrointestinal**
**lymphatic cancer**
Less than 1 year	269 (63.7)	12.81 ± 4.04	0.158	27.49 ± 3.68	0.048	33.54 ± 2.91	0.106
1–3 years	114 (27.0)	12.84 ± 4.18		27.31 ± 3.62		33.32 ± 3.65	
More than 3 years	39 (9.2)	14.03 ± 5.23		28.85 ± 4.10		34.59 ± 3.42	
**Have received chemotherapy treatment**
No	132 (31.3)	12.77 ± 4.86	0.332	27.93 ± 3.81	0.161	33.41 ± 3.81	0.458
Yes	290 (68.7)	13.00 ± 3.88		27.40 ± 3.68		33.65 ± 2.86	
**Currently undergoing chemotherapy**
**treatment**
No	195 (46.2)	13.58 ± 4.34	< 0.001	28.11 ± 3.72	0.001	33.66 ± 3.53	0.099
Yes	227 (53.8)	12.37 ± 4.01		27.10 ± 3.67		33.50 ± 2.86	
**During chemotherapy, have healthcare**
**providers provided health education**
No	16 (3.8)	9.50 ± 3.88	0.022	27.25 ± 3.07	0.935	32.50 ± 2.71	0.111
Yes	406 (96.2)	13.07 ± 4.16		27.58 ± 3.75		33.62 ± 3.20	

The first row represents the overall mean scores of knowledge, attitude, and practice. P-values were derived from t-tests or ANOVA for continuous variables and chi-square tests for categorical variables.

SD, standard deviation; KAP, knowledge, attitudes, and practices; GI, gastrointestinal.

### Knowledge, attitude, and practice dimensions

The mean knowledge score among participants was 12.93 ± 4.21 (Table 1). The statement regarding the avoidance of bad habits, cessation of smoking and drinking, adherence to a regular schedule, and ensuring adequate rest during chemotherapy was most recognized as “Very familiar” by 45.7% of respondents. In contrast, the information about the high incidence of complications like intestinal obstruction, perforation, and acute peritonitis in GI lymphoma patients undergoing chemotherapy, which can be life-threatening, was least familiar to participants, with 21.6% indicating “Not familiar” (Supplementary Table 1). Analysis of the factors associated with knowledge revealed that participants from households with monthly incomes below 2,000 RMB exhibited significantly lower knowledge scores (OR = 0.370, *P* = 0.001). Previous exposure to chemotherapy correlated with higher knowledge scores (OR = 2.361, *P* = 0.003), whereas current chemotherapy treatment was linked to lower scores (OR = 0.279, *P* < 0.001; Table 2).

**Table 2:** Univariable and multivariable logistic regression for knowledge.

**Knowledge**	**Univariate analysis**		**Multivariate analysis**	
	**OR (95% CI)**	***P***	**OR (95% CI)**	***P***
**Gender**
Male	0.810 (0.499, 1.297)	0.387		
Female				
**Age, years old**
18–49				
50–64	0.580 (0.264, 1.190)	0.152		
65–74	0.638 (0.291, 1.306)	0.236		
≥75	0.540 (0.215, 1.330)	0.181		
**BMI, kg/m** ^2^
< 18.5	0.695 (0.344, 1.467)	0.322		
18.5–23.9				
24.0–27.9	1.039 (0.606, 1.813)	0.891		
≥28.0	0.904 (0.427, 2.056)	0.799		
**Residence**
Rural				
Urban	1.964 (1.235, 3.124)	0.004	1.318 (0.730, 2.379)	0.360
**Marital status**
Married				
Unmarried/divorced/widowed	3.904 (1.134, 24.543)	0.067	3.480 (0.728, 16.644)	0.118
**Education**
Primary school or below	0.484 (0.238, 0.941)	0.038	0.975 (0.387, 2.458)	0.957
Middle school	0.572 (0.279, 1.120)	0.112	0.865 (0.372, 2.007)	0.735
High school/technical secondary school	2.196 (0.827, 6.520)	0.129	2.564 (0.863, 7.614)	0.090
Associate or above				
**Monthly income, yuan**
< 2,000	0.284 (0.165, 0.477)	< 0.001	0.370 (0.201, 0.679)	0.001
2,000–5,000				
>5,000	0.790 (0.363, 1.830)	0.564	0.724 (0.299, 1.754)	0.474
**Medical insurance**
Have social health insurance				
No social health insurance	1.487 (0.384, 9.786)	0.612		
**Diabetes**
No				
Yes	1.296 (0.693, 2.582)	0.436		
**Hyperlipidemia**
No				
Yes	0.964 (0.591, 1.601)	0.886		
**Time of diagnosis of gastrointestinal lymphatic cancer**
Less than 1 year				
1–3 years	1.692 (0.978, 3.040)	0.068	1.337 (0.724, 2.468)	0.354
More than 3 years	1.128 (0.528, 2.630)	0.767	0.942 (0.390, 2.274)	0.894
**Have received chemotherapy treatment**
No				
Yes	1.525 (0.943, 2.446)	0.082	2.361 (1.337, 4.169)	0.003
**Currently undergoing chemotherapy treatment**
No				
Yes	0.323 (0.192, 0.529)	< 0.001	0.279 (0.159, 0.491)	< 0.001
**During chemotherapy, have healthcare providers provided**
**health education**
No				
Yes	1.138 (0.312, 3.355)	0.827		

Reference groups: female (gender); 18–49 years (age); 18.5–23.9 kg/m^2^ (BMI); rural (residence); married (marital status); associate degree or above (education); 2,000–5,000 RMB (monthly income); having social health insurance (insurance status); no (diabetes); no (hyperlipidemia); less than 1 year (time since diagnosis); no (history of chemotherapy); no (currently undergoing chemotherapy); no (health education by providers).

OR, odds ratio; CI, confidence interval; KAP, knowledge, attitudes, and practices; GI, gastrointestinal.

The mean attitude score was 27.57 ± 3.72 (range: 7–35), indicating generally positive attitudes toward chemotherapy (Table 1). Most participants (74.4%) agreed that adhering to chemotherapy is very important for the treatment of the disease, with an additional 94 (22.3%) strongly agreeing. A total of 317 participants (75.1%) agreed that when adverse reactions occur, they would seek help from a doctor in a timely manner, and that the guidance from healthcare professionals increases their confidence in the treatment (Supplementary Table 2). Knowledge levels significantly influenced attitudes (OR = 1.397, *P* < 0.001). Participants aged 65–74 years tended to show less positive attitudes compared to younger groups, although this association was not statistically significant (OR = 0.541, *P* = 0.149). Lower income (< 2,000 RMB monthly) was associated with less positive attitudes (OR = 0.500, *P* = 0.013). Interestingly, those without social health insurance showed significantly more positive attitudes (OR = 19.714, *P* = 0.021), although this finding should be interpreted cautiously due to the small sample size of uninsured participants (Table 3).

**Table 3:** Univariable and multivariable logistic regression for attitude.

**Attitude**	**Univariate analysis**		**Multivariate analysis**	
	**OR (95% CI)**	***P***	**OR (95% CI)**	***P***
Knowledge	1.429 (1.312, 1.556)	< 0.001	1.397 (1.278, 1.527)	< 0.001
**Gender**
Male				
Female	0.672 (0.450, 0.998)	0.050	0.785 (0.472, 1.306)	0.351
**Age, years old**
18–49				
50–64	0.590 (0.321, 1.063)	0.083	0.885 (0.392, 2.000)	0.770
65–74	0.446 (0.244, 0.798)	0.007	0.541 (0.235, 1.246)	0.149
≥75	0.587 (0.276, 1.239)	0.163	0.747 (0.273, 2.045)	0.571
**BMI, kg/m** ^2^
< 18.5	1.475 (0.770, 2.897)	0.247		
18.5–23.9				
24.0–27.9	1.010 (0.644, 1.585)	0.967		
≥28.0	0.896 (0.461, 1.740)	0.744		
**Residence**
Rural				
Urban	1.057 (0.708, 1.576)	0.786		
**Marital status**
Married				
Unmarried/divorced/widowed	2.157 (0.954, 5.336)	0.076	1.134 (0.362, 3.553)	0.829
**Education**
Primary school or below	0.439 (0.243, 0.777)	0.005	0.938 (0.388, 2.269)	0.887
Middle school	0.368 (0.204, 0.650)	0.001	0.714 (0.319, 1.598)	0.412
High school/technical secondary school	0.585 (0.298, 1.137)	0.115	0.576 (0.247, 1.346)	0.203
Associate or above				
**Monthly income, yuan**
< 2,000	0.424 (0.277, 0.646)	< 0.001	0.500 (0.289, 0.866)	0.013
2,000–5,000				
>5,000	1.564 (0.849, 2.973)	0.160	1.031 (0.444, 2.391)	0.944
**Medical insurance**
Have social health insurance				
No social health insurance	9.977 (1.915, 183.210)	0.028	19.714 (1.563, 248.667)	0.021
**Diabetes**
No				
Yes	1.607 (0.948, 2.776)	0.082	1.533 (0.793, 2.962)	0.204
**Hyperlipidemia**
No				
Yes	1.050 (0.690, 1.599)	0.821		
**Time of diagnosis of gastrointestinal lymphatic cancer**
Less than 1 year				
1–3 years	1.003 (0.647, 1.557)	0.988	0.880 (0.516, 1.499)	0.637
More than 3 years	2.381 (1.168, 5.173)	0.021	1.963 (0.783, 4.921)	0.150
**Have received chemotherapy treatment**
No				
Yes	0.826 (0.544, 1.248)	0.365		
**Currently undergoing chemotherapy treatment**
No				
Yes	0.526 (0.356, 0.776)	0.001	0.718 (0.446, 1.155)	0.172
**During chemotherapy, have healthcare providers**
**provided health education**
No				
Yes	0.893 (0.314, 2.442)	0.826		

Reference groups: female (gender); 18–49 years (age); 18.5–23.9 kg/m^2^ (BMI); rural (residence); married (marital status); associate degree or above (education); 2,000–5,000 RMB (monthly income); having social health insurance (insurance status); no (diabetes); no (hyperlipidemia); less than 1 year (time since diagnosis); no (history of chemotherapy); no (currently undergoing chemotherapy); no (health education by providers).

OR, odds ratio; CI, confidence interval; KAP, knowledge, attitudes, and practices.

The mean practice score was 33.57 ± 3.19 (range: 8–40), suggesting good adherence to recommended practices. Three hundred and twenty-two participants (76.3%) reported that they often would report symptoms like palpitations or dizziness to healthcare professionals promptly, with an additional 95 (22.5%) always doing so. A high proportion (317, 75.1%) often persisted with treatment during the first and second cycles of chemotherapy, even if it was very painful, with 89 (21.1%) always doing so. Additionally, 165 (39.1%) participants always avoided bad habits, quit smoking and drinking, maintained a regular schedule, and ensured a normal and healthy routine during the treatment period (Supplementary Table 3). Both knowledge and attitude scores were found to influence practice scores positively. Higher knowledge scores were associated with better practice scores (OR = 1.235, *P* < 0.001). However, the impact of attitude on practice, while positive, was not statistically significant in the multivariate analysis (OR = 1.041, *P* = 0.543; Table 4).

**Table 4:** Univariable and multivariable logistic regression for practice.

**Practice**	**Univariate analysis**		**Multivariate analysis**	
	**OR (95% CI)**	***P***	**OR (95% CI)**	***P***
Knowledge	1.264 (1.136, 1.405)	< 0.001	1.235 (1.101, 1.385)	< 0.001
Attitude	1.159 (1.042, 1.290)	0.007	1.041 (0.916, 1.183)	0.543
**Gender**
Male	0.636 (0.294, 1.287)	0.224		
Female				
**Age, years old**
18–49				
50–64	0.438 (0.123, 1.230)	0.150		
65–74	1.012 (0.267, 3.229)	0.985		
≥75	0.378 (0.094, 1.329)	0.139		
**BMI, kg/m** ^2^
< 18.5	0.693 (0.276, 1.986)	0.459		
18.5–23.9				
24.0–27.9	1.141 (0.530, 2.611)	0.744		
≥28.0	4.371 (0.875, 79.442)	0.155		
**Residence**
Rural				
Urban	1.357 (0.679, 2.658)	0.377		
**Marital status**
Married				
Unmarried/divorced/widowed	0.778 (0.255, 3.386)	0.693		
**Education**
Primary school or below	0.979 (0.322, 2.746)	0.968		
Middle school	0.747 (0.253, 1.976)	0.571		
High school/technical secondary school	0.535 (0.171, 1.566)	0.259		
Associate or above				
**Monthly income, yuan**
< 2,000	0.608 (0.286, 1.250)	0.182		
2,000–5,000				
>5,000	0.877 (0.315, 2.830)	0.811		
**Medical insurance**
Have social health insurance				
No social health insurance	1.091 (0.204, 20.221)	0.934		
**Diabetes**
No				
Yes	2.377 (0.824, 10.070)	0.160		
**Hyperlipidemia**
No				
Yes	1.643 (0.765, 3.944)	0.229		
**Time of diagnosis of gastrointestinal lymphatic cancer**
Less than 1 year				
1–3 years	1.418 (0.648, 3.443)	0.407		
More than 3 years	0.936 (0.339, 3.311)	0.907		
**Have received chemotherapy treatment**
No				
Yes	1.894 (0.953, 3.716)	0.064	1.695 (0.826, 3.477)	0.150
**Currently undergoing chemotherapy treatment**
No				
Yes	1.182 (0.604, 2.313)	0.623		
**During chemotherapy, have healthcare providers**
**provided health education**
No				
Yes	2.446 (0.542, 8.038)	0.178		

Reference groups: female (gender); 18–49 years (age); 18.5–23.9 kg/m^2^ (BMI); rural (residence); married (marital status); associate degree or above (education); 2,000–5,000 RMB (monthly income); having social health insurance (insurance status); no (diabetes); no (hyperlipidemia); less than 1 year (time since diagnosis); no (history of chemotherapy); no (currently undergoing chemotherapy); no (health education by providers).

OR, odds ratio; CI, confidence interval; KAP, knowledge, attitudes, and practices.

### Structural equation modeling analysis

**Figure 1:**
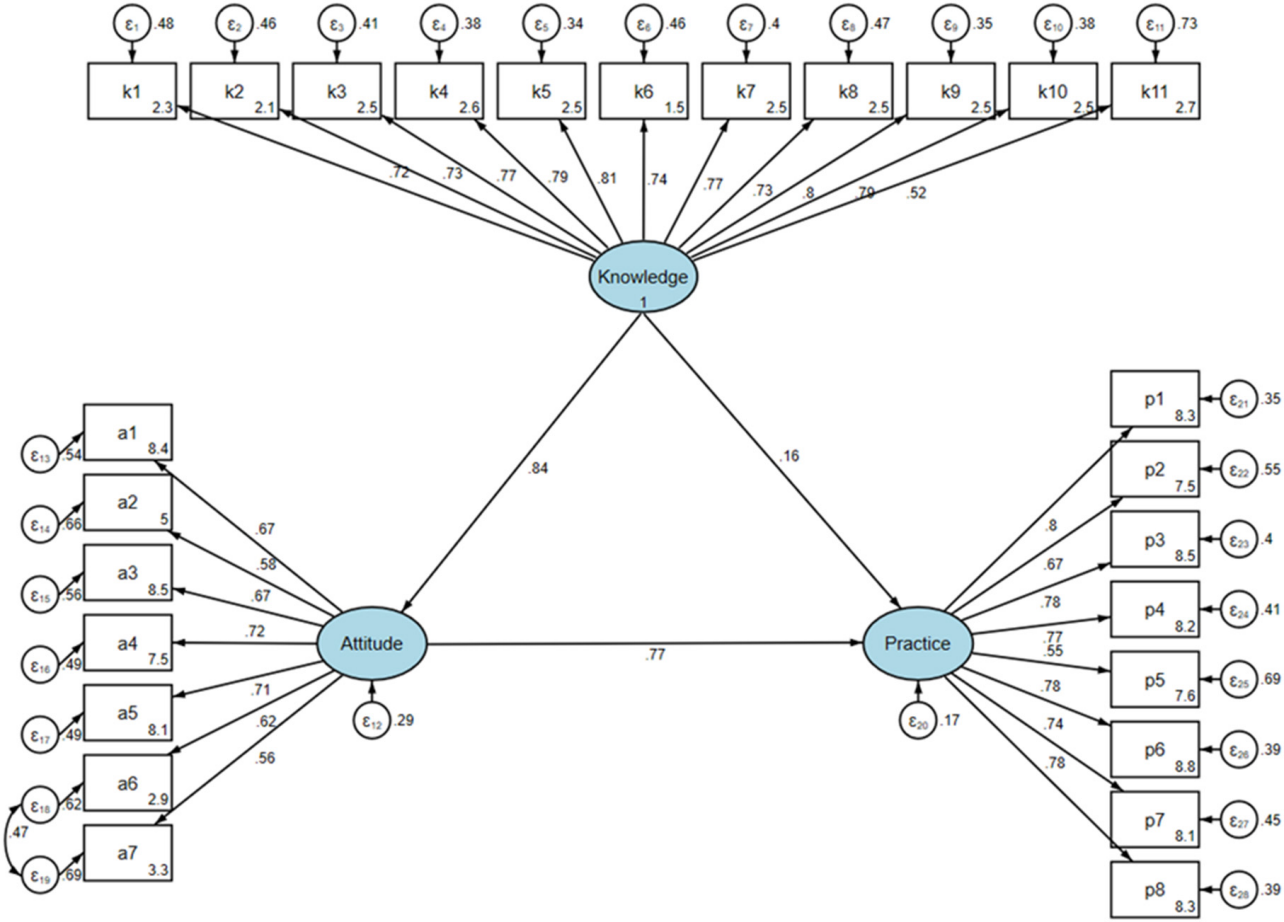
SEM results.

The SEM indicates that knowledge exerts a significant total effect on attitudes (β = 0.844, *P* < 0.001), attitudes significantly influence practices directly (β = 0.770, *P* < 0.001), and knowledge exerts a significant total effect on practices (β = 0.810, *P* < 0.001). Further, knowledge affects practices both directly (β = 0.161, *P* = 0.043) and indirectly (β = 0.649, *P* < 0.001; Table 5, Figure 1). The SEM fitting effect, as supported by Supplementary Table 4, validates the model's accuracy and reliability, with all fit indices indicating a good overall fit of the model to the data.

**Table 5:** SEM results.

**Model paths**	**Total effects**		**Direct Effect**		**Indirect effect**	
	β **(95% CI)**	***P***	β **(95% CI)**	***P***	β **(95% CI)**	***P***
**Asum**
Ksum	0.844 (0.802, 0.886)	< 0.001	0.844 (0.802, 0.886)	< 0.001		
**Psum**
Asum	0.770 (0.618, 0.922)	< 0.001	0.770 (0.618, 0.922)	< 0.001		
Ksum	0.810 (0.769, 0.852)	< 0.001	0.161 (0.005, 0.316)	0.043	0.649 (0.507, 0.792)	< 0.001

β represents standardized path coefficients.

SEM, structural equation modeling; CI, confidence interval; KAP, knowledge, attitudes, and practices.

## Discussion

This study provides comprehensive insights into the KAP of patients with GI lymphoma regarding chemotherapy adverse reactions and related care. The results highlight the impact of significant urban–rural differences and factors such as prior chemotherapy experience on patients' understanding and management of their treatment. The development and evaluation of targeted educational programs to improve patient awareness of chemotherapy and recommended care measures may lead to better treatment attitudes and practices.

Previous studies have not directly evaluated the KAP of patients with GI lymphoma on the side effects of chemotherapy, but some research teams have evaluated the KAP of cancer patients. The study found that most patients had positive attitudes toward chemotherapy, but their knowledge and practice level were limited. Patients with higher levels of education had significantly higher scores in KAP (20).

In the knowledge dimension, patients exhibited a moderate level of understanding regarding chemotherapy-related information. Notably, there were significant disparities in knowledge across various aspects. Approximately half of patients (45.7%) were “very familiar” with the importance of maintaining a healthy lifestyle during chemotherapy, indicating a high degree of awareness regarding lifestyle management. Conversely, a significant number of patients demonstrated a lack of awareness about serious risks. The most concerning finding was that 21.6% of patients were “not familiar” with the fact that about 40% of patients with GI lymphoma undergoing chemotherapy experience potentially life-threatening complications such as intestinal obstruction, intestinal perforation, and acute peritonitis. This lack of awareness about severe complications is particularly worrisome. This finding aligns with previous research by Olver et al. (16), which indicated that a substantial proportion of chemotherapy patients may be unaware of information about serious side effects and potential adverse events associated with their treatment. Two thirds of patients say they seek immediate medical attention for a high fever (67%), but only 41% seek immediate medical attention for bleeding or bruising. The gap in knowledge regarding severe complications is especially critical, as it may lead to delayed recognition of symptoms and potentially impact patient outcomes. Several factors influence the level of patient knowledge. Urban residents had higher knowledge scores compared to rural residents, consistent with the health disparities between urban and rural areas in China reported by Fang et al. (21). Patients with lower monthly household incomes exhibited significantly lower knowledge scores, supporting the findings of Meng et al. (22) regarding the impact of socioeconomic status on access to medical services. More recent national and regional studies within the past 5 years also report that urban–rural disparities in healthcare access and utilization remain evident in China, despite ongoing improvements in medical infrastructure and insurance coverage (23–25). These investigations found that rural residents continue to experience lower healthcare utilization rates and reduced access to medical services compared with urban populations. Patients with prior experience of chemotherapy had higher knowledge scores, whereas those currently undergoing chemotherapy had lower scores. This pattern suggests the accumulation of knowledge through experience but also potential barriers to information acquisition during active treatment. The lower knowledge scores among patients currently undergoing chemotherapy are particularly concerning, as this is when accurate information about side effects and complications is most crucial. Similar findings have been reported, indicating that many patients undergoing chemotherapy lack adequate understanding of which adverse reactions require prompt medical attention (16). These findings highlight the urgent need for comprehensive patient education programs, especially focusing on potentially severe complications of chemotherapy. Evidence from previous research also supports the effectiveness of structured educational interventions; a study demonstrated that targeted nursing education significantly improved patients' ability to recognize and manage chemotherapy-related symptoms (8). Healthcare providers should ensure that all patients, regardless of their socioeconomic status or place of residence, receive clear and thorough information about the risks associated with their treatment. Special attention should be given to patients, perhaps through regular educational sessions or the provision of easily accessible, understandable information materials. Furthermore, the significant knowledge gap regarding life-threatening complications suggests that current patient education strategies may be inadequate in conveying the seriousness of certain side effects. This calls for a reevaluation of how risk information is communicated to patients, possibly incorporating more effective communication strategies or visual aids to ensure better understanding and retention of critical information.

The attitude dimension results indicate generally positive attitudes. The majority recognized the importance of adhering to chemotherapy for disease treatment and indicated they would promptly seek medical help when experiencing adverse reactions. This positive attitude is consistent with findings from a study in northern Tanzania, where cancer patients maintained positive attitudes toward chemotherapy despite limited awareness (26). The study found that knowledge levels significantly influenced attitudes toward chemotherapy. This finding supports previous research demonstrating a statistically significant relationship between side effect knowledge and chemotherapy attitudes, particularly among more educated cancer patients (27). Age and income levels also influenced patient attitudes. The older age group showed fewer positive attitudes, aligning with research by Peters et al. (28) indicating that older cancer survivors have less positive attitudes toward self-management and e-health. The community can organize health social education activities for the elderly on a regular basis to encourage the elderly to pay attention to health indications or cancer-related information for prevention and treatment, to reduce anxiety and worry related to chemotherapy. Patients with lower monthly incomes demonstrated fewer positive attitudes, consistent with findings from El Rhazi et al. (29) on the relationship between socioeconomic status and cancer awareness. So the government in addition to need to pay attention to the essence of medical resource allocation imbalance, also should pay attention to people in the rural medical knowledge acquisition.

The practice dimension results suggest that patients generally adhere well to medical recommendations. A high proportion of patients persisted with treatment during the initial chemotherapy cycles. This positive practice behavior aligns with findings from Jansen et al. (27), who reported that cancer patients in the Netherlands initially had positive attitudes toward self-management practices (30).

Finally, the interactions among KAP of this study reveal a complex interplay that significantly influences patient outcomes. Knowledge was found to have both direct and indirect effects on practices. The direct effect of knowledge on practices indicates that better-informed patients are more likely to adhere to recommended practices. However, the indirect effect of knowledge on practices through attitudes was substantially stronger. This suggests that knowledge primarily influences practices by first shaping patients' attitudes. The strong relationship between knowledge and attitudes underscores how critical it is for patients to understand their condition and treatment. Furthermore, attitudes showed a significant direct effect on practices, highlighting that positive attitudes toward chemotherapy are crucial for encouraging adherence to treatment protocols. These findings emphasize the importance of comprehensive patient education programs that not only impart knowledge but also address attitudes, as both elements are vital in shaping patients' practices during chemotherapy.

It is essential to acknowledge the limitations of this study. The single-center design may restrict the generalizability of the results. The sample size, while sufficient for the current analysis, may limit our ability to perform detailed subgroup analyses or fully reveal complex multivariate interactions. In addition, the exclusion of very elderly patients, individuals with certain comorbidities, and those with cognitive or psychiatric conditions may limit the generalizability of the findings to all patients with gastrointestinal lymphoma. Furthermore, including patients who had not yet started chemotherapy may have introduced bias into the assessment of practice. These limitations highlight the need for future research to employ larger, multi-center studies and more advanced statistical methods.

## Conclusion

In conclusion, patients with GI lymphoma demonstrated moderate knowledge, positive attitudes, and good practices regarding chemotherapy adverse reactions and care. Targeted interventions to improve knowledge, especially among rural and lower-income patients, may enhance overall attitudes and practices toward chemotherapy management.

## Data Availability

The original contributions presented in the study are included in the article/Supplementary material, further inquiries can be directed to the corresponding author.
