# Supplementary material for: Knowledge, attitudes, and practices of chemotherapy adverse reactions and care among patients with gastrointestinal lymphoma
Source: Front Med (Lausanne). 2026 Jan 23;12:1722022. doi: 10.3389/fmed.2025.1722022 (PMC12875986; doi:10.3389/fmed.2025.1722022)
Supplement: Supplementary file 1 [file Supplementary_file_1.docx]

**Supplementary table 1 Knowledge dimension responses**

| **Knowledge** | Very familiar | Somewhat familiar | Not familiar |
| --- | --- | --- | --- |
| **1. Gastrointestinal primary lymphoma is a type of extranidal lymphoma, with non-Hodgkin lymphoma being the most common.** | 84(19.9%) | 310(73.5%) | 28(6.6%) |
| **2. Gastrointestinal lymphoma is effectively treated with chemotherapy. Even if surgery thoroughly removes the tumor, recurrence and distant metastasis can occur. Therefore, postoperative chemotherapy should be considered routine treatment.** | 82(19.4%) | 302(71.6%) | 38(9%) |
| **3. Bone marrow suppression is one of the most common toxic reactions to chemotherapy drugs. Its main manifestations include a reduction in all three lines of hematopoietic cells, significant decreases in white blood cells, platelet reduction, and coagulation disorders. During chemotherapy, patients should check their blood counts at least 1-2 times per week.** | 89(21.1%) | 317(75.1%) | 16(3.8%) |
| **4. Nausea and vomiting are common adverse gastrointestinal reactions during chemotherapy, which may be due to the stimulation of the central vomiting receptor area by chemotherapy drugs or acute gastric mucosal injury.** | 84(19.9%) | 325(77%) | 13(3.1%) |
| **5. If brown or bloody fluid or yellow bile is found in the vomit, stop the medication immediately and observe to avoid serious complications such as acute gastritis, gastric ulcers, and gastric bleeding.** | 95(22.5%) | 313(74.2%) | 14(3.3%) |
| **6. About 40% of patients with gastrointestinal lymphoma undergoing chemotherapy experience complications such as intestinal obstruction, intestinal perforation, and acute peritonitis, which can threaten the patient's life.** | 79(18.7%) | 252(59.7%) | 91(21.6%) |
| **7. During chemotherapy, oral mucosal erosion and ulcers may occur, so it is important to maintain oral hygiene.** | 108(25.6%) | 302(71.6%) | 12(2.8%) |
| **8. Chemotherapy drugs such as doxorubicin and vincristine can cause varying degrees of dermatitis and significant hair loss.** | 80(19%) | 323(76.5%) | 19(4.5%) |
| **9. Decreased appetite is one of the common symptoms of gastrointestinal lymphoma, which may cause weight loss and psychological distress.** | 106(25.1%) | 303(71.8%) | 13(3.1%) |
| **10. During chemotherapy, patients should follow a reasonable diet, starting with a full liquid diet, then a semi-liquid diet, and gradually transitioning to a regular diet.** | 85(20.1%) | 320(75.8%) | 17(4%) |
| **11. During chemotherapy, patients should avoid bad habits, quit smoking and drinking, maintain a regular schedule, and ensure normal healthy rest times.** | 193(45.7%) | 220(52.1%) | 9(2.1%) |

**Supplementary table 2 Attitude dimension responses**

| **Attitude** | Strongly agree | Agree | Neutral | Disagree | Strongly disagree |
| --- | --- | --- | --- | --- | --- |
| **1. You believe that adhering to chemotherapy is very important for the treatment of the disease.** | 94(22.3%) | 314(74.4%) | 12(2.8%) | 1(0.2%) | 1(0.2%) |
| **2. You believe that the adverse reactions caused by treatment can be alleviated or avoided.** | 89(21.1%) | 234(55.5%) | 75(17.8%) | 23(5.5%) | 1(0.2%) |
| **3. You believe that understanding the adverse reactions caused by treatment before starting treatment is very important.** | 89(21.1%) | 315(74.6%) | 16(3.8%) | 2(0.5%) | 0 (0%) |
| **4. In order to treat gastrointestinal lymphoma, you are willing to accept the adverse reactions and physical discomfort caused by the treatment.** | 95(22.5%) | 302(71.6%) | 20(4.7%) | 4(0.9%) | 1(0.2%) |
| **5. When adverse reactions occur, you will seek help from a doctor in a timely manner. The guidance and comfort from healthcare professionals make you confident in the treatment.** | 88(20.9%) | 317(75.1%) | 13(3.1%) | 3(0.7%) | 1(0.2%) |
| **6. Gastrointestinal discomfort and various adverse reactions caused by chemotherapy reduce your compliance and confidence in the treatment.** | 89(21.1%) | 150(35.5%) | 41(9.7%) | 139(32.9%) | 3(0.7%) |
| **7. Gastrointestinal discomfort and various adverse reactions caused by chemotherapy make you feel anxious and distressed.** | 79(18.7%) | 197(46.7%) | 31(7.3%) | 113(26.8%) | 2(0.5%) |

**Supplementary Table 3 Practice dimension responses**

| **Practice** | Always | Often | Sometimes | Few | Never |
| --- | --- | --- | --- | --- | --- |
| **1. You will thoroughly understand the adverse reactions caused by chemotherapy from healthcare professionals.** | 95(22.5%) | 315(74.6%) | 9(2.1%) | 2(0.5%) | 1(0.2%) |
| **2. During the treatment period, you will monitor your stool color, volume, and consistency, and watch for signs of fresh blood or vomiting blood.** | 81(19.2%) | 317(75.1%) | 16(3.8%) | 7(1.7%) | 1(0.2%) |
| **3. During the first and second cycles of chemotherapy, even if it is very painful, you will persist.** | 89(21.1%) | 317(75.1%) | 15(3.6%) | 0 (0%) | 1(0.2%) |
| **4.After the third cycle of chemotherapy, you strictly follow the dietary transition from a full liquid diet to a semi-liquid diet, and then gradually to a regular diet.** | 88(20.9%) | 323(76.5%) | 7(1.7%) | 2(0.5%) | 2(0.5%) |
| **5.During the treatment period, you avoid bad habits, quit smoking and drinking, maintain a regular schedule, and ensure a normal and healthy routine.** | 165(39.1%) | 244(57.8%) | 10(2.4%) | 2(0.5%) | 1(0.2%) |
| **6.If you experience symptoms like palpitations or dizziness, you will report them to healthcare professionals promptly.** | 95(22.5%) | 322(76.3%) | 2(0.5%) | 2(0.5%) | 1(0.2%) |
| **7.During the treatment period, you can overcome conditions such as hair loss, nausea, vomiting, and constipation.** | 92(21.8%) | 317(75.1%) | 10(2.4%) | 1(0.2%) | 2(0.5%) |
| **8.Although you feel distressed by the adverse drug reactions caused by chemotherapy, you will not give up the treatment.** | 104(24.6%) | 303(71.8%) | 14(3.3%) | 0 (0%) | 1(0.2%) |

**Supplementary Table 4 SEM fitting effect**

| **Indicators** | **Reference** | **Results** |
| --- | --- | --- |
| RMSEA | <0.08Good | 0.074 |
| SRMR | <0.08Good | 0.056 |
| TLI | >0.8Good | 0.834 |
| CFI | >0.8Good | 0.849 |
